# Enablers Supporting the Implementation of Knowledge Management in the Healthcare of Pakistan

**DOI:** 10.3390/ijerph15122816

**Published:** 2018-12-10

**Authors:** Jawad Karamat, Tong Shurong, Naveed Ahmad, Abdul Waheed, Kashif Mahmood

**Affiliations:** 1 School of Management, Northwestern Polytechnical University, Xi’an 710072, Shannxi, China; stong@nwpu.edu.cn (T.S.); naveedahmad@mail.nwpu.edu.cn (N.A.); waheed_2506@mail.nwpu.edu.cn (A.W.); 2 Department of Management Science, Bahria University, Islamabad 44220, Pakistan; kashifmahmood81@gmail.com

**Keywords:** enablers, Knowledge management, KM, healthcare, health care, Interpretive structural modeling, ISM, MICMAC, Pakistan

## Abstract

Knowledge is considered to be an important resource; it is the source of competitive advantage. However, if knowledge is managed well with Knowledge Management (KM), then it becomes a source for sustainable competitive advantage for organizations. If KM is implemented in an organization, it would improve the organizational competitiveness, performance, and productivity, and facilitate the efficient use of resources. Due to intense competition in the global market, many organizations are moving towards the adoption of KM. The healthcare sectors of many developed countries have moved towards the implementation of KM because it can improve the procuring of knowledge from ongoing activities by the effective use of data repositories. Developing countries have now realized the potential and benefits of KM adoption. Pakistan is one of the developing countries that have recently shown an inclination towards the adoption of KM in its healthcare sector to improve performance of its healthcare. This study was composed of two main research phases. Firstly, the enablers of KM were reviewed from earlier studies. Secondly, interpretive structural modeling (ISM) and MICMAC (Cross-Impact Matrix Multiplication Applied to Classification) techniques were used to show the interrelationships between KM enablers and driving and dependence power of each enabler. The application of ISM and MICMAC technique shows that policy incentive, long-term strategic planning, Information Technology (IT), and alignment of KM efforts with business strategy are the main enablers of KM adoption in the healthcare of Pakistan. Focusing on the identified enablers will help in the implementation of KM. Policy incentives can work as a catalyst to promote KM adoption in the healthcare of Pakistan.

## 1. Introduction

Knowledge management (KM) is a process used by organizations and individuals to locate, store, retrieve, share, adapt, and use of knowledge that supports the organization to achieve its objectives [1,2]. Organizations have now realized that if they want to survive in the rapidly changing environment, their knowledge must be well used [3,4,5]. KM and organizational learning give organizations an ability to adapt the rapidly changing business environment [6,7]. If an organization wants to be efficient and effective in the way they operate, then it is critical to adopt KM [5,8].

Currently, knowledge management has gained the attention of many scholars and researchers. There have been many discussions about the criticality of KM in various fields such as supply chain [9], engineering industries [10], telecommunication [11], healthcare [12], etc. which believe that the transfer of knowledge is the source of gaining a sustainable competitive advantage [13,14]. Knowledge is considered to be the main resource within an organization [15]. Many researchers have identified that there are two types of knowledge, tacit and explicit. Explicit knowledge represents everything that can be readily articulated, codified, accessed verbalized, and can be easily transmitted to another, in the form of books, articles, manuals, databases etc. Tacit knowledge is difficult to pass on to another because it is acquired through practice and personal experience. KM supports the conversion of tacit knowledge to explicit, which results in knowledge creation and improves the performance of organizations [16].

Organizations that are linked to knowledge creation possess the ability to improve their performance and better use of intermediate knowledge for further innovations [17]. KM has been implemented in many organizational and managerial processes of the healthcare sector of developed countries [18]. The healthcare sector is a late adopter of KM as compared to other business sectors [19]; the adoption of KM in the healthcare sector benefits the patients by providing them with better service, the employees by learning new and better methods, and organization by getting more business. Orzano et al. [20] claimed that KM at a national level, between health care providers, would significantly improve (medical) practice performance and benefit both patient and doctor.

Several developed countries have moved towards the adoption of KM in the healthcare sector [21,22] to improve their healthcare performance. Developing countries are now moving towards the adoption of KM, but it is at slow pace. Iran, among developing countries, has done the most work towards KM adoption [12]. The government of Pakistan is now looking for methods to improve its healthcare sector, so it is considering the adoption of KM in its healthcare sector.

Pakistan is facing several problems and challenges in its healthcare sector. The World Health Organization (WHO) has stated that Pakistan is facing a double burden of disease (BoD), as 7.6% of the population is infected with hepatitis B and C. The prevalence of tuberculosis is fifth highest in the world, and it is estimated that 4.3% of it is drug resistant. A large area of the population suffers from malaria; it is the second most commonly reported disease and, despite it being curable, many people die of it. Pakistan accounts for 81% of the malaria deaths globally [23]. The rate of people infected with HIV is about 0.1% and is growing, 77% of the individuals were infected due to unscreened blood transfusion. Pakistan has a very high infant mortality rate; it is estimated that 400,000 infants die within the first year due to acute respiratory tract infection, viral hepatitis, malaria, diarrhea, dysentery, scabies, goiter, hepatitis, tuberculosis, etc. There are many patients with non-communicable diseases, injuries, and mental health issues. Pakistan has the seventh highest population of diabetes (figures from the World Health Organization [24]). The continuous increase in the population is resulting in increased accidents and many injuries. The healthcare sector of Pakistan must deal with many patients daily, and the outpatient department (OPD) is always overcrowded. There is often a problem of management, resources, and performance [25]. Pakistan is now considering the introduction of KM to help in resolving these problems to a certain extent.

There are several advantages that KM may offer to the healthcare sector. Keeping problems of the healthcare sector of Pakistan in mind, a scientific study identifying the enablers to promote KM adoption needs to be conducted. Therefore, The aim of this study is to identify the enablers that support KM implementation in the healthcare sector of Pakistan. The current study was undertaken for the following reasons: firstly, there is a scarcity of research on KM adoption in the healthcare of developing countries, especially in Pakistan. Currently, KM is at the infancy stage in Pakistan. Secondly, a detailed literature review was conducted to study the enablers that support the effective implementation of KM in the healthcare sector; these enablers further were discussed with a panel of experts, and they used a multiple-criteria decision-making (MCDM) approach to analyze the enablers of KM adoption in the context of Pakistan. Finally, the shortlisted enablers were analyzed to reveal the interrelationship between them using the ISM and MICMAC approach.

The structure of this paper is as follows: Section 1 has the introduction of the study, Section 2 gives the literature review, Section 3 describes the research methodology, Section 4 discusses the results, and Section 5 gives the conclusion.

## 2. Literature Review

### 2.1. Knowledge Management

Globalization has made knowledge a key resource for achieving competitive advantage [26]. Knowledge adds value to an organization through its contribution to products, processes, and people, while KM transforms information, data, and intellectual assets into enduring value by identifying useful knowledge for management actions, KM gives the organization a sustainable competitive advantage [27].

The organizations have acknowledged KM as a source of sustainable competitive advantage and have started spending heavily for its implementation. A KM adoption survey conducted in healthcare, manufacturing, retail/wholesale, utilities, telecommunication, financial services, and other sectors showed that 43% of the organizations had a KM initiative in place. Many organizations are considering KM as an option to transform their business for the sake of sustainable development [28]. KM is positively affecting many industries especially the management consultancies and health care whose primary product is knowledge.

The healthcare sector organizations have slowly moved towards KM adoption, to improve its performance [21,22]. Healthcare Information & Management Systems Society (HIMSS) stated KM in health care is “aligning people, processes, data, and technologies to optimize information, collaboration, expertise, and experience in order to drive organizational performance and growth.”

### 2.2. KM in the Healthcare of Pakistan

Pakistan is among developing countries, a part of South Asia with a population of about 201 million and growing rapidly at a rate of 1.9%. Pakistan despite being the second largest economy in South Asia with a gross domestic product (GDP) of 988.2 billion [29] is having trouble with its healthcare sector. Pakistan has continuously increased the budget of healthcare over the years [30] but still is unable to cope with the rapid growth and increase in patients on a national level, and the performance is below average.

The government of Pakistan realizes the complexity of improving its healthcare sector. To deal with this problem, over the years, Pakistan has presented many policies and strategies such as National Health Policy (2001); Medium Term Development Framework (2005–2010); and Poverty Reduction Strategy Papers (2001–2009). Despite having developed these policies, the performance of the healthcare sector was not improving, but the government persisted on developing new policies [31]. The government of Pakistan in 2001 developed the National Health Vision setting targets for 2025; it took 15 years for approval by both federal and provincial government, and was finally approved in 2016 [32]. Pakistan has also signed many international agreements such as Millennium Development Goals (2000) [33] and Sustainable Development Goals (2016): 17 goals to transform our world [34], by United Nations. The government of Pakistan spent Rs4.06 trillion since 2012–2013, to achieve the Millennium Development Goals (MDGs) in health, education, social welfare, and other areas but it failed to achieve the goals [35]. Turning to the Sustainable Development Goals (SDGs), the task facing the country is even greater. The government of Pakistan is now looking for new methods to improve its healthcare performance.

The implementation of KM can improve the performance of healthcare greatly. KM helps in the recording of knowledge; this knowledge can be shared to improve performance [36] of the healthcare sector, and it also helps in effective decision-making [37]. Since the healthcare industry is a knowledge-generating sector, it is important to record the knowledge that is used on a daily basis [5].

### 2.3. Enablers of KM in Healthcare

A detailed literature review was done to identify the enablers of KM. Several enablers were identified from various previous studies. Karamitri et al. [2] stated four enablers, the commitment of management, the development of transparent work flows, trust among the employees, employee empowerment, and identification of the knowledge broker. Chang et al. [38] mentioned that developing a transparent workflow, taking continuous customer feedback, and establishing a good relationship with the customers as enablers of KM. Hojabri et al. [12] identified seven enablers, supporting organizational culture, supportive management, good information technology infrastructure, effective knowledge management strategy, continuous performance management, good training and education, evaluation of process and activities as enablers of KM. Out of these seven training and education was considered to be most critical. Gibbs et al. [39] revealed that the government policies also prove to be enablers in the implementation of KM. Kothari et al. [19] mentioned six main enablers of KM, organizational culture, organization structure, management support, identification of knowledge champion, a proper KM design or framework and good training. Kulkarni et al. [40] stated, leadership, interaction between coworkers, learning from existing knowledge, taking feedback from customer and recruiting of experienced workers for effective implementation of KM as the key enablers. Pee and Kankanhalli [41] identified three main enablers for KM, Information technology, the worker has to be motivated, and skillful in the implementation of KM, or to higher skillful worker, out of these three Information Technology was considered more critical. Chang et al. [42] revealed that, an interactive learning environment, a good knowledge filtration system, effective customer feedback system and Information technology can significantly help in the implementation of KM. Alavi and Leidner [43] focused only on one enabler, they mentioned that knowledge must be properly arranged and categorized to avoid knowledge loss and information overflow.

From these studies the enablers most relevant to the healthcare of Pakistan are given in Table 1.

## 3. Research Methodology

This study uses interpretive structural modeling (ISM) and MICMAC technique for analysis of KM enablers. To apply this technique, the research methodology has been divided into three steps. The steps are explained below.

Step 1: A comprehensive literature review was conducted on the enablers of KM in the healthcare sector. These enablers were identified by studying peer-reviewed publications. These publications were searched using several databases such as Google Scholar, Scopus, Web of Science, Emerald, Taylor & Francis and Science Direct. The keywords used to draw out these researches were: “Knowledge management”, “healthcare” and “enablers”. A total of 170 papers from over 60 journals came up using these keywords. Two steps were performed to narrow down to the most relevant papers. First, the duplication of papers was removed. Secondly, the abstracts were studied of the remaining papers and the irrelevant papers were removed. Finally, 43 papers from 30 journal and three conferences were left. There were some popular journals among the list of journals considered for this paper they are (number of papers), Journal of Knowledge Management (6 papers), Sustainability (5 papers), Journal of Management in Engineering (3 papers), International Journal of Management Science and Engineering Management (3 papers), Journal of Management Information Systems (3 papers), Expert Systems with Applications (2 paper) and Behavior & Information Technology (1 paper).

Step 2: In the literature review, several papers were studied. Some papers mentioned 15 enablers while some suggested 5. A total of 25 enablers were considered for this study as a result of the literature review. To shortlist the enablers relevant to Pakistan a panel of experts was selected. The selection of experts was done randomly on the basis of convenience sampling. Initially, 30 healthcare workers (Doctors, Admin staff, nursing staff) were contacted via email and telephone, their information was available on their hospital websites. After frequent contact via emails and calls, two medical doctors, one hospital Chief Operating Officer, two Head nurses agreed to participate in the study. Using the same method five professors were contacted, two of them responded with a willingness to participate. To include an expert from the government side frequent face to face meetings were held to convince them to participate, eventually, one Assistant Director from the Department of Health Khyber Pakhtunkhwa (KPK) agreed. Finally, an expert panel of eight experts was made; all the experts considered for this study are highly qualified and knowledgeable with a minimum of 10 years’ experience in their respective fields. The main questions experts were asked is as follows:

Q1: Which enablers are helping the implementation of KM in the healthcare sector of Pakistan?

The selected panel of experts got together to address the above-mentioned question and to shortlist the enablers according to the healthcare of Pakistan. Following the procedure adopted by Waqas, et al. [63], experts were asked to provide response in a preliminary survey based on their knowledge and practical experience. The results of preliminary survey were compiled with the help of a facilitator and a summary of results was further sent to experts to add or delete the enablers. After completing three repetitions and consensus of experts, 18 enablers were shortlisted. The shortlisting was done with the help of a 5-point scale. The geometric mean was taken, if the enabler was not above 3 it was rejected. 

Step 3: Once the final set of enablers have been identified they will be analyzed using the ISM and MICMAC technique. In ISM, with the help of experts’ opinion and practical knowledge, contextual relationships between the enablers are identified, and a hierarchical relationship between them is made. The MICMAC analysis helps in the analysis and explanation of the enablers with the help of driving and dependence power.

**Figure 1:**
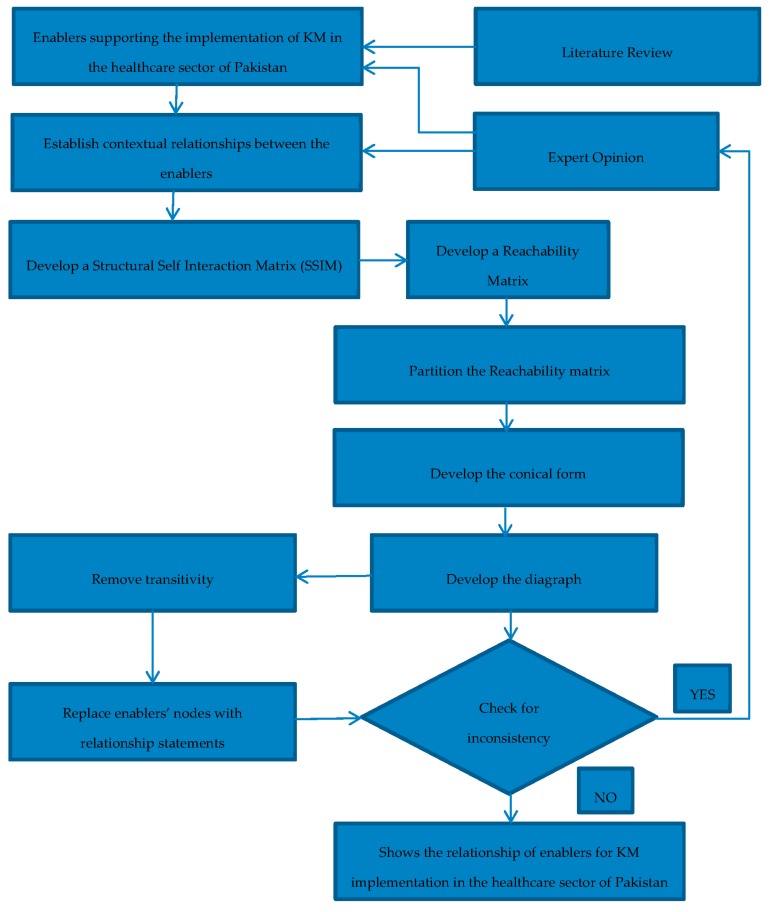
Research methodology.

After brainstorming and detailed discussions with experts their responses were recorded. This study shows the interrelationship and the hierarchical structure of the enablers that are helping in the implementation of KM in the healthcare sector of Pakistan to gain a sustainable competitive advantage. The step-by-step research methodology for this study is given in Figure 1.

### 3.1. Interpretive Structural Analysis (ISM)

ISM gets its roots from Structural Modeling (SM), which was created by J.N. Warfield (1974) [64]. It is an interactive learning process in which a panel of experts get together to determine the direct and indirect relationship that exists between the variables. A model is formed which shows the complex relationships and interrelationships that exist between the variables [65]. This model helps in understanding the root cause. The ISM technique is preferred for several reasons, firstly, there are many problems that need to be solved in researches. These problems can be complex and difficult to understand because there may be a presence of many interrelationships that exist between the variables [66]. ISM determines these relationships and interrelationships with the help of experts. Secondly, structural equation modeling (SEM) is a statistical technique that helps in testing variables that affect a problem, but it fails where there are interrelationships between the variables. The ISM technique here is used for the creation of an initial model [64]. Thirdly, ISM is a group learning process which helps in making the unclear and poorly made system into a well-defined model [67]. Fourthly, there is no need to consult prior works to apply ISM, and finally ISM can be applied in various fields, such as healthcare, education, supply chain, etc. [68].

The ISM model is used by many experts, to show the order and complex interrelationship among the variables, and to present the variables in the form of a model. Singh et al. [69] used ISM in analyzing barriers to KM adoption in engineering industry of India. Anantatmula and Kanungo [70] used the ISM-MICMAC technique to analyze the enablers in business organizations for effective implementation of KM. Kumar and Sharma [71] used the ISM and MICMAC technique to identify and analyze the barriers to growth of rural health care of India. Karamat et al. [72] used the ISM-MICMAC technique to identify and analyze the barriers to KM in the healthcare sector of Pakistan. To apply the ISM technique some well-defined steps have to be followed according to the guideline of Kannan, et al. [73]. The steps are as follows.

Step 1 Identifying the enablers of KM implementation in the healthcare sector through comprehensive literature review.
Step 2 Shortlist the enablers with the help of expert opinion, using 5-point scale and geometric mean. The variables with a value of less than 3 are rejected.
Step 3 Developing the Structural Self-Interaction Matrix (SSIM), to show the pairwise relationship between the variables.
Step 4 Developing the Initial Reachability Matrix (IRM) by digitizing the SSIM with 1 and 0.
Step 5 Incorporating the transitivity, the hidden interrelationships are explained, if A is related to B, B is related to C, then A and C must also be related in some way,
Step 6 The Final Reachability Matrix (FRM) is developed, in this all the transitivity is removed,
Step 7 Level partitions are made; this is done by giving the variables different levels,
Step 8 Making the ISM model, the model is made on the basis of level partitions, the level at the top is level 1, as the level increases so does its criticality, and finally
Step 9 The MICMAC analysis is done. It is done with the help of driving and dependence power.

The detail of the steps are given below.

#### 3.1.1. Structural Self-Interaction Matrix (SSIM)

To develop the SSIM, experts have to develop contextual relationships among the various enablers [74]. The 18 enablers are written vertically (Y-axis) from 1 to 18 and horizontally on the (X-axis) in the table. The cell Eij shows the relationship between Ei (Y-axis) and Ej (X-axis). The relationship is shown with the help of four symbols, V, A, X, and O.

- V shows that enabler i helps to achieve or influence enabler j;
- A shows that enabler j helps to achieve or influence enabler i;
- X shows that enabler i and enabler j help to achieve or influence each other; and
- O shows that there is no relationship between enabler i and enabler j

The SSIM table of this study is given below in Table 2.

#### 3.1.2. Initial Reachability Matrix (IRM)

IRM is developed after SSIM. The IRM shows the relationship between the enablers in binary format. It is converted into binary format by replacing V, A, X, and O symbols with 1 and 0. The conversion is done following these rules.

- If in the SSIM the relationship (i, j) is represented with the V symbol, then the entry (i, j) is 1 and (j, i) is 0;
- If in the SSIM the relationship (i, j) is represented with the A symbol, then the entry (i, j) is 0 and (j, i) is 1;
- If in the SSIM the relationship (i, j) is represented with the X symbol, then the entry (i, j) is 1 and (j, i) is also 1; and
- If in the SSIM the relationship (i, j) is represented with the O symbol, then the entry (i, j) is 0 and (j, i) is also 0;

The rules are as shown in Table 3.

By applying the rules given in Table 3, Table 4 was derived; an example of conversion is that in SSIM table the relationship of enabler 3 and enabler 4 is represented with a V. Therefor in the IRM the entry (3, 4) is 1, and the entry in (4, 3) is 0.

#### 3.1.3. Final Reachability Matrix (FRM)

After IRM, FRM is developed. To develop the FRM, the transitivity is removed from IRM, this is an important feature of the ISM model. Transitivity can be defined as if, A is related to B, B is related to C, then A and C must also be related in some way. After removing the transitivity, the hidden relationship is denoted by 1* in Table 5.

Table 5 has rows and columns; the rows are reachability and the columns are the antecedent. The reachability (driving power) can be defined as a set of variables that will help in achieving/reaching that variable. The reachability set consists of variables that are represented by “1” in that row. The sum of those 1’s gives the driving power of the variable. The antecedents (dependence power) can be defined as a set consisting of variables which can reach a certain variable. The antecedent set consists of variables that are represented by “1” in that column. The sum of those 1’s gives the dependence power of the variable. The variables that are present in both reachability (r) and antecedent set (a) belong to intersection set (int).

(1) int=(r∩a)

The driving and dependence power of enablers of this study is given in Table 5.

#### 3.1.4. Level Partitions

After the making of FRM, the level partition is done. From the FRM table, the reachability and antecedent sets for each enabler is derived. With the help of these sets, the intersection set is made. The three sets are then put in a table form (Table 6, Table 7, Table 8, Table 9, Table 10, Table 11 and Table 12). To do the level partition several iterations are developed.

To determine the levels, the intersection set is compared with the reachability set, if they are the same then that makes a level. Once the level has been determined then the number assigned to that enabler is removed from the existing table. For example, in Table 6, the intersection and reachability of enabler 6 and 11 is same, hence they will form level 1 and they will be removed from the next table. This step is continued until all the enablers have been assigned a level. The levels start from the top and slowly move down. The top level has the least effect, and the bottom level is the most critical. There are 18 enablers considered for this paper, with the help of level partition they were divided into seven levels. These level partitions helped in the development of the ISM model. The level partitions of, enablers of KM implementation in the healthcare sector of Pakistan are given below in the following tables, from 6–12.

All the levels are shown in Table 13.

#### 3.1.5. ISM Model

**Figure 2:**
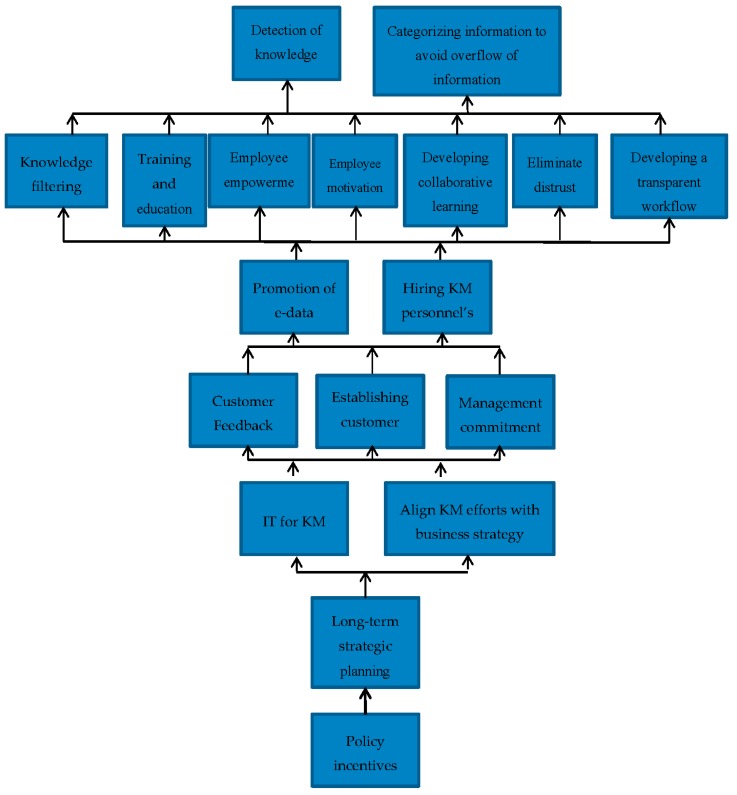
Interpretive Structural Modeling model for enablers of knowledge management implementation in the healthcare sector of Pakistan.

Once the level partition is done then the next step is to make the conical matrix. The conical matrix shows the levels in the proper order, from level one to level seven (as per this study) as shown in the previous Table 13. After the conical matrix, the digraph is made. The digraph is a set of nodes that shows the interrelationship between them with help of arrows. The levels are shown with help of level partition and the relationship on the basis of FRM. After the digraph is made it is then converted into an ISM model. The nodes of the digraph are replaced with the enabler description. The enablers of level one are less critical; as the level goes higher, they become more critical. The ISM model of this paper is shown in Figure 2.

### 3.2. MICMAC Analysis

The ISM model helps to understand the interrelationships between the various enablers, whereas MICMAC (Cross-Impact Matrix Multiplication Applied to Classification) helps in understanding the extent to which one enabler is influenced by the other. The MICMAC analysis is performed with the help of driving and dependence power. The driving power is calculated by adding all the 1’s in the respective rows of the enabler from the FRM in Table 6. The driving power shows the influence of that enabler on other enablers. The dependence power is calculated by adding all the 1’s in the respective column of the enabler from the FRM Table 6. The dependence power shows the extent to which the enabler is affected by the other enablers.

The enablers are then plotted on a graph depending on their driving and dependence power. The driving power is represented on the Y-axis whereas the dependence power is on the X-axis. The graph is divided into four clusters. These clusters are autonomous enabler, dependent enabler, linkage enabler, and independent enabler. They are explained below.

- Autonomous enabler: the enablers in Quadrant I are autonomous enablers, these enablers have weak driving and dependence power. These enablers do not have much effect on other enablers. These enablers are disconnected and remain extraneous.
- Dependent enablers: the enablers in Quadrant II are dependent enablers; these enablers have weak driving but strong dependence power. These enablers are highly influenced by other enablers. If an action is taken on the influential enablers it will affect these enablers as well.
- Linkage enablers: the enablers in Quadrant III are linkage enablers; these enablers have high driving and dependence power. These enablers are very volatile, any action taken on one enabler will affect the other and itself in the process.
- Independent enablers: the enablers in Quadrant IV are independent enablers; these enablers have a strong driving but weak dependence power. If any action is taken on these enablers they will affect other enablers that are dependent on these enablers.

**Figure 3:**
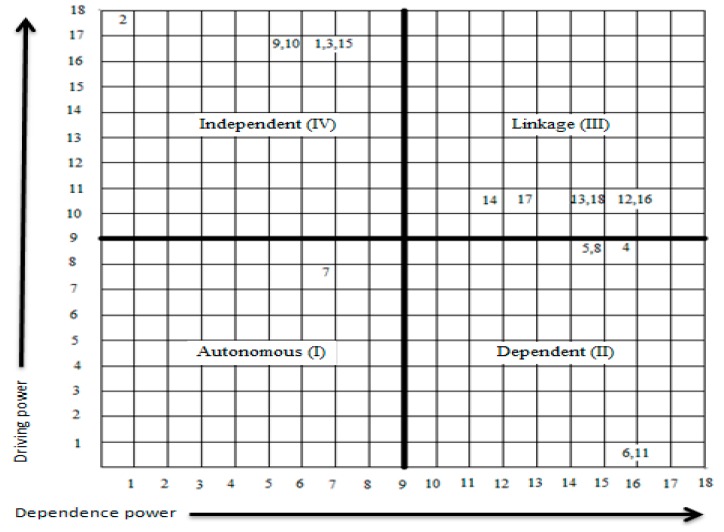
Driving v/s dependence power using MICMAC analysis.

The enablers in the independent and linkage quadrants are the Key enablers, because of their strong driving power. The MICMAC analysis of the enablers that help in the implementation of KM in the healthcare sector of Pakistan are given below in Figure 3.

According to the MICMAC analysis of this study, there is only one enabler in Quadrant I (Autonomous) it is enabler 7. It has been placed here because its driving power is 8 and dependence power is 7. All the other enablers were placed following the same example. The enablers of Quadrant II (Dependent) are 4, 5, 6, 8 and 11. The key enablers are placed in Quadrant III (Linkage) and IV (Independent). Enablers of Quadrant III are 12, 13, 14, 16, 17, and 18, and Quadrant IV are 1, 2, 3, 9, 10 and 15.

## 4. Results and Discussion

Over the past few years, KM has gained a considerable amount of importance, experts consider it as a key to sustainable competitive advantage [75]. Many organizations are looking towards the implementation of KM to improve their competitiveness [76], including the healthcare organizations [19]. KM brings improvement into the business process, promotes better coordination with other departments and staff, and protects from knowledge loss. Since the healthcare sector is a knowledge-rich area [77] a lot of knowledge is being generated by the healthcare sector, this knowledge needs to be stored, which can be done with the help of KM.

To protect Pakistan healthcare sector from knowledge loss, this study was conducted. Pakistan is now looking towards the implementation of KM in its healthcare sector, to make it more sustainable and help in providing better healthcare service. Since KM is in its initial stages in Pakistan there is insufficient research in this area; the current study will try to identify the enablers in Pakistan that will help with the implementation of KM in the healthcare sector of Pakistan. This study uses the ISM and MICMAC technique to identify the enablers and their interrelationships. The results of the study are as follows.

1. Autonomous enablers: According to current study one enabler lies in Quadrant I, it has weak driving and dependence power. This enabler does not have much impact and is disconnected and remain extraneous. It is enabler 7 (establishing customer relationship management (CRM)). This enabler least helps in supporting the implementation of KM in the healthcare sector of Pakistan. However, the presence of one autonomous enabler shows that the other enablers selected by this research help significantly.
2. Dependent enablers: the enablers in Quadrant II have weak driving but strong dependence power. According to current study 5 of the enablers lie in this quadrant, which include enabler 4 (Developing transparent workflow), enabler 5 (Eliminate distrust), enabler 6 (Detection of knowledge brokers), enabler 8 (Developing collaborative learning atmosphere), and enabler 11 (Categorizing information to avoid overflow of information).
3. Linkage enablers: the enablers in Quadrant III have strong driving and dependence powers, they are considered to be among the Key enablers. These enablers help in affecting the other enablers. If one of these enablers is used to help, then the other enablers will also provide some assistance. According to the current study 12 of the enablers lie in Quadrant III; they are enabler 12 (Employee motivation), enabler 13 (Employee empowerment), enabler 14 (Promotion of e-data), enabler 16 (Training and education), enabler 17(Hiring KM personnel’s) and enabler 18 (Knowledge filtering).
4. Independent enabler: the enablers in Quadrant IV have strong driving but weak dependence power. These are the main (Key) enablers; they support the implementation of KM in the healthcare sector of Pakistan the most. According to the current study 6 enablers lie in this category, they are enabler 1 (Management commitment), enabler 2 (Policy incentives), enabler 3 (Long-term strategic planning), enabler 9 (Align KM efforts with business strategy), enabler 10 (IT for KM), and enabler 15 (Customer Feedback).

The ISM model shows the variables in the form of hierarchy, the most critical at the lower level and the variables with minor impact at the upper level. In the current study, the ISM model shows the enablers in the form of a hierarchical structure that is helping in KM implementation in the healthcare sector of Pakistan. According to the current study, the enabler that could help the most in KM implementation in the healthcare sector of Pakistan is “policy incentive” (enabler 2). The government of Pakistan plays a major role in the healthcare sector since it has one of the largest healthcare infrastructures in the world [78]. The healthcare sector is administered by the federal and the provincial government, whereas the districts are responsible for the implementation. Pakistan has initiated many national programs such as community health workers program, to provide communities with primary health care facilities and outreach activities. Pakistan has realized that it faces many problems in its healthcare service delivery [24], due to this several policies were developed, National Health Policy (Health Sector Reform) (2001), Medium Term Development Framework (2005–2010), and Health Millennium Development Goals (2015) [31]. Pakistan has also increased the budget that is spent on healthcare sector over time [30]. This is high time to implement KM in the healthcare sector to further improve the performance. When the policy is favorable to healthcare sector then long-term strategic planning should be done to improve the KM implementation.

Long-term strategic planning (enabler 3) is compulsory for the implementation of KM. Perera and Peiró [79] mentioned that strategic planning is compulsory for all types of organizations, especially the healthcare sector organizations. With the help of strategic planning, the healthcare sector organizations can meet their short, medium, and long-term goals, and they can transform for a better future. In the long-term strategic planning the vision, mission, and values must be clearly defined. The Ministry of Health (Pakistan) has realized the importance of strategic planning and has made a Nation Health Vision [32]. In this document, the vision, mission, and values of the healthcare sector have been clearly stated, and Pakistan wants to improve its health standards. The hospitals in the private sector are facing great competition from the public sector. The private healthcare organizations are comparatively better when it comes to strategic planning, but there is a lot of room for improvement. Both the public and private sector can gain considerably from the implementation of strategic planning for KM. The public, as well as private-sector health organizations, are looking forward to improving and towards KM adoption. Hence this proves to be an enabler for KM. Effective long-term strategic planning gives rise to more enablers.

Effective strategic planning gives rise to the alignment of KM efforts with business strategy (enabler 9) and Information Technology (IT) for KM (enabler 10). In large organizations, many strategic plans coexist, all these plans must be consistent and aligned with each other in order to succeed. Greiner et al. [80] mentioned that for successful implementation of KM there must be an alignment with business strategy. Similarly, the strategy for the implementation of KM must be aligned with that of the Ministry of Health [79] for the public sector healthcare sector organizations. The Ministry of Health (Pakistan) is looking for new methods to improve its performance in the healthcare sector; this will prove to be an effective enabler for KM implementation. The long-term strategies to implement KM will require technology in hospitals since KM depends on IT (enabler 10). Acharyulu [81] mentioned that IT is necessary for KM in healthcare to effectively meet the global healthcare challenges and future trends. The Ministry of Health has increased its spending on technology over time to cope with the current changes. It is trying to get more technology to improve IT and healthcare. In 2018, $3.04 billion were spent on healthcare sector which is 31.75% more compared to last year. The amount spent on technology is $457.1 million and by 2020 it is expected to be about $537.5 million. The private sector has always been a step ahead of the public sector when it comes to technology. Hence technology is an important enabler for KM implementation in the healthcare sector of Pakistan and it further gives rise to other enablers.

Alignment of KM efforts with business strategy (enabler 9) and IT for KM (enabler 10) give rises to management commitment (enabler 1), establishing CRM (enabler 7), and customer feedback (enabler 15). To implement any KM strategy or system the support of management is crucial [82]. The executives should also be involved to build a KM initiative to ensure its success [83,84] in the healthcare sector. An influential executive who supports KM would highly influence its implementation [85,86]. In the public healthcare sector (Pakistan) organization, an effort should be made to motivate the management (administration) to help and take part in KM implementation. Due to the large infrastructure of the public healthcare sector organizations, it will not be easy, but it can be possible through developing policy guidelines. The private-sector (Pakistan) healthcare organizations are more active and involved with its customers and can easily implement KM. The private-sector hospitals, to improve their performance and strengthen their customer base, are moving towards CRM. The CRM in KM focuses on customer knowledge, it helps in providing the customer better service [52]. Other than CRM, a customer (the customer may be internal or external) feedback system must be established, feedback helps the KM process [40] by making changes to KM system to make it more efficient and user-friendly. The private sector is quick to take feedback of its customers; they are always looking for a chance to improve their business. These organizations listen to their physicians and other staff (internal customers) [42] who make complaints or suggestions if they face problems as well as patients (external customers). The public sector does not pay attention to CRM; they are already overcrowded with patients [87,88], and there is no proper feedback system. The introduction of these enablers will assist the public healthcare sector organizations for effective KM implementation. These enablers further give rise to other enablers.

The above-aforementioned enablers give rise to the promotion of e-data (enabler 14) and hiring KM personnel’s (enabler 17). When the management is committed, and the healthcare sector organizations are willing to have a feedback system then they can promote e-data and the healthcare sector organizations can hire new KM experts to implement KM in the hospitals. Promotion of e-data (enabler 14) and hiring KM personnel’s (enabler 17) further give rise to more enablers such as developing transparent workflow (enabler 4), elimination of distrust (enabler 5), developing a collaborative learning atmosphere (enabler 8), employee motivation (enabler 12), employee empowerment (enabler 13), training and education (enabler 16), and knowledge filtering (enabler 18).

Finally, the enablers that are least helpful but must be considered are, the detection of knowledge brokers (enabler 6) and categorizing information to avoid overflow of information (enabler 11). There are many enablers that have been identified in the healthcare sector of Pakistan. The relevant authorities and experts should get together and deal with these enablers to effectively implement KM in the healthcare sector of Pakistan to improve the quality of patient care and welfare.

## 5. Conclusions

Business organizations are now moving towards managing their knowledge to gain a sustainable competitive advantage over competitors. The organizations that manage their knowledge well are likely to perform better in the future [75]. Healthcare sector organizations rely heavily on information and knowledge from inside and outside. Outside knowledge is gained from published literature and professional conferences attended by doctors. Inside information comes from patient care, knowledge, and skills of workers. The combination of these two helps in creating new knowledge [89]. The storing of knowledge in hospitals is critical for two reasons. Firstly, it helps in improving the performance of the hospital by keeping patients records [90], and secondly, some healthcare sector institutions in the US keep knowledge to protect itself from lawsuits [91]. The Ministry of Health (Pakistan) is trying to find new methods and ways to improve its healthcare service; this study proposes KM as a method to improve health services by implementing it in the healthcare sector.

Many healthcare organizations in developed countries are adopting KM [22] since it is the source of gaining a sustainable competitive advantage. Developing countries are now also looking towards KM implementation to improve their performance [12]. Pakistan is also trying to adopt new techniques to improve healthcare performance. Due to this the current study considers KM as an option to improve healthcare performance, since no research is present regarding KM implementation in the healthcare sector of Pakistan.

The aim of this study is to identify and analyze the contextual relationships between the enablers using ISM technique and to develop a hierarchy of enablers in the healthcare sector of Pakistan. After identifying KM enablers from a literature review and feedback from a panel of eight experts, 18 enablers were shortlisted in this study. By implementing ISM technique the driving and dependence powers were determined. The ISM model of this study shows the enablers in a hierarchical structure from most critical to the least critical.

The 18 enablers identified by this study were partitioned into seven levels. According to the findings of this study, the key enabler that forms the foundation of the structure is policy incentive (enabler 2) and other enablers including long-term strategic planning (enabler 3), alignment of KM efforts with business strategy (enabler 9), and IT for KM (enabler 10) are also critical. These are independent barriers, having strong driving power but weak dependence power. The Government of Pakistan (Ministry of Health) plays a vital role in the healthcare sector since it mainly provides the healthcare services; favorable policies must be developed in order to implement KM in the healthcare of Pakistan.

The contribution of this study is that it clearly identifies the enablers and how they are related to each other with the help of the ISM and MICMAC approaches. This study will give a clear idea of how to effectively implement KM in the healthcare sector of Pakistan, and where to focus first. The current study shows that if the base enabler, policy incentive (enabler 2), is focused on, it will lead towards more enablers that will support for successful implementation of KM. It is now up to the relevant authorities (federal and provincial governments) and entities (hospitals (public/private), staff, unions’ etc.) to implement KM in the healthcare sector. KM will provide a sustainable competitive advantage and help to compete globally against international rivals.

There are certain limitations that apply to the current study. Since this study uses the ISM and MICMAC approach, it is highly dependent on the feedback of experts. There is a chance of bias among the experts selected, and the analysis is based on the knowledge that they possess at the time of conducting current study. Over time they can change their opinion. The enablers considered by the current study number 18. In relation to the enablers that help in the implementation of KM in the healthcare sector of Pakistan, in future, other studies can consider more or fewer enablers, according to the situation of the sector and country.

In the future, total interpretive structural modeling (TISM) may be used for this study, since ISM and MICMAC analysis does not have the capability to illustrate the interpretive logic of dominance/interaction among enablers. Furthermore, the ISM methodology only helps in showing the relationships and establishes the initial model; it does not assign weights or statistically validate the enablers. Therefore it is suggested to apply SEM to the current study to test and validate the model.

## Figures and Tables

**Table 1:** Literature review on enablers of knowledge management implementation in the healthcare sector.

No	Enabler	Reference
1	Management commitment	[2,41,44]
2	Policy incentives	[39,45,46]
3	Long-term strategic planning	[12,47,48]
4	Developing a transparent workflow	[2,38,49]
5	Eliminate distrust	[2,12,41,50]
6	Detection of knowledge brokers	[2,19,51]
7	Establishing customer relationship management (CRM)	[38,52]
8	Developing collaborative learning atmosphere	[12,40,42,53,54]
9	Alignment of knowledge management (KM) goals and objectives	[12,47,55]
10	IT for KM	[2,38,41,42,56]
11	Categorizing information to avoid overflow of information	[12,43]
12	Employee motivation	[2,41,44]
13	Employee empowerment	[2]
14	Promotion of e-data	[2,57]
15	Customer Feedback	[40,42,53]
16	Training and education	[12,47,58,59]
17	Hiring KM personnel’s	[40,41,60]
18	Knowledge filtering	[2,42,61,62]

**Table 2:** Structural Self-Interaction Matrix for enablers of knowledge management implementation in the healthcare sector of Pakistan.

Enablers		1	2	3	4	5	6	7	8	9	10	11	12	13	14	15	16	17	18
1	Management commitment	---	A	X	V	V	V	V	V	V	V	O	O	O	O	X	V	V	O
2	Policy incentives			V	O	O	O	V	O	O	V	V	V	O	V	O	V	V	O
3	Long-term strategic planning				V	O	V	V	V	V	V	O	O	V	V	X	V	V	O
4	Developing a transparent workflow					X	V	O	O	O	O	V	X	X	O	A	A	O	O
5	Eliminate distrust						O	O	A	O	O	O	V	A	O	O	A	O	O
6	Detection of knowledge brokers							O	A	A	O	O	A	O	O	O	A	O	O
7	Establishing customer relationship management (CRM)								O	A	A	O	O	O	O	X	O	O	O
8	Developing collaborative learning atmosphere									A	O	O	X	V	O	O	A	O	O
9	Align KM efforts with business strategy										X	V	V	O	V	V	V	V	V
10	IT for KM											V	O	O	V	O	V	V	V
11	Categorizing information to avoid overflow of information												A	A	O	O	A	A	A
12	Employee motivation													A	O	A	X	O	V
13	Employee empowerment														O	O	A	O	O
14	Promotion of e-data															O	X	A	V
15	Customer Feedback																V	V	O
16	Training and education																	O	V
17	Hiring KM personnel’s																		X
18	Knowledge filtering																		

**Table 3:** Rules of IRM.

Structural Self-Interaction Matrix	Initial Reachability Matrix (IRM)	
Element (i, j)	Element (i, j)	Element (j, i)
V	1	0
A	0	1
X	1	1
O	0	0

**Table 4:** Initial reachability matrix for enablers of knowledge management implementation in the healthcare sector of Pakistan.

	1	2	3	4	5	6	7	8	9	10	11	12	13	14	15	16	17	18
1	1	0	1	1	1	1	1	1	1	1	0	0	0	0	1	1	1	0
2	1	1	1	0	0	0	1	0	0	1	1	1	0	1	0	1	1	0
3	1	0	1	1	0	1	1	1	1	1	0	0	1	1	1	1	1	0
4	0	0	0	1	1	1	0	0	0	0	1	1	1	0	0	0	0	0
5	0	0	0	1	1	0	0	0	0	0	0	1	0	0	0	0	0	0
6	0	0	0	0	0	1	0	0	0	0	0	0	0	0	0	0	0	0
7	0	0	0	0	0	0	1	0	0	0	0	0	0	0	1	0	0	0
8	0	0	0	0	1	1	0	1	0	0	0	1	1	0	0	0	0	0
9	0	0	0	0	0	1	1	1	1	1	1	1	0	1	1	1	1	1
10	0	0	0	0	0	0	1	0	1	1	1	0	0	1	0	1	1	1
11	0	0	0	0	0	0	0	0	0	0	1	0	0	0	0	0	0	0
12	0	0	0	1	0	1	0	1	0	0	1	1	0	0	0	1	0	1
13	0	0	0	1	1	0	0	1	0	0	1	1	1	0	0	0	0	0
14	0	0	0	0	0	0	0	0	0	0	0	0	0	1	0	1	0	1
15	1	0	1	1	0	0	1	0	0	0	0	1	0	0	1	1	1	0
16	0	0	0	1	1	1	0	1	0	0	1	1	1	1	0	1	0	1
17	0	0	0	0	0	0	0	0	0	0	1	0	0	1	0	0	1	1
18	0	0	0	0	0	0	0	0	0	0	1	0	0	0	0	0	1	1

**Table 5:** Final reachability matrix for enablers of knowledge management implementation in the healthcare sector of Pakistan.

	1	2	3	4	5	6	7	8	9	10	11	12	13	14	15	16	17	18	Driving Power
**1**	1	0	1	1	1	1	1	1	1	1	1*	1*	1*	1*	1	1	1	1*	17
**2**	1	1	1	1*	1*	1*	1	1*	1*	1	1	1	1*	1	1*	1	1	1*	18
**3**	1	0	1	1	1*	1	1	1	1	1	1*	1*	1	1	1	1	1	1*	17
**4**	0	0	0	1	1	1	0	1*	0	0	1	1	1	0	0	1*	0	1*	9
**5**	0	0	0	1	1	1*	0	1*	0	0	1*	1	1*	0	0	1*	0	1*	9
**6**	0	0	0	0	0	1	0	0	0	0	0	0	0	0	0	0	0	0	1
**7**	1*	0	1*	1*	0	0	1	0	0	0	0	1*	0	0	1	1*	1*	0	8
**8**	0	0	0	1*	1	1	0	1	0	0	1*	1	1	0	0	1*	0	1*	9
**9**	1*	0	1*	1*	1*	1	1	1	1	1	1	1	1*	1	1	1	1	1	17
**10**	1*	0	1*	1*	1*	1*	1	1*	1	1	1	1*	1*	1	1*	1	1	1	17
**11**	0	0	0	0	0	0	0	0	0	0	1	0	0	0	0	0	0	0	1
**12**	0	0	0	1	1*	1	0	1	0	0	1	1	1*	1*	0	1	1*	1	11
**13**	0	0	0	1	1	1*	0	1	0	0	1	1	1	1*	0	1*	1*	1*	11
**14**	0	0	0	1*	1*	1*	0	1*	0	0	1*	1*	1*	1	0	1	1*	1	11
**15**	1	0	1	1	1*	1*	1	1*	1*	1*	1*	1	1*	1*	1	1	1	1*	17
**16**	0	0	0	1	1	1	0	1	0	0	1	1	1	1	0	1	1*	1	11
**17**	0	0	0	1*	1*	1*	0	1*	0	0	1	1*	1*	1	0	1*	1	1	11
**18**	0	0	0	1*	1*	1*	0	1*	0	0	1	1*	1*	1*	0	1*	1	1	11
**Dependence power**	7	1	7	16	15	16	7	15	6	6	16	16	15	12	7	16	13	15	

After removing the transitivity, the hidden relationship is denoted by 1*.

**Table 6:** Level partition for enablers—Iteration 1.

Enablers	Reachability Sets	Antecedent Set	Intersections	Levels
1	1, 3, 4, 5, 6, 7, 8, 9, 10, 11, 12, 13, 14, 15, 16, 17, 18	1, 2, 3, 7, 9, 10, 15	1, 3, 7, 9, 10, 15	
2	1, 2, 3, 4, 5, 6, 7, 8, 9, 10, 11, 12, 13, 14, 15, 16, 17, 18	2	2	
3	1, 3, 4, 5, 6, 7, 8, 9, 10, 11, 12, 13, 14, 15, 16, 17, 18	1, 2, 3, 7, 9, 15	1, 3, 7, 9, 15	
4	4, 5, 6, 8, 11, 12, 13, 16, 18	1, 2, 3, 4, 5, 7, 8, 9, 10, 12, 13, 14, 15, 16, 17, 18	4, 5, 8, 12, 13, 16, 18	
5	4, 5, 6, 8, 11, 12, 13, 16, 18	1, 2, 3, 4, 5, 8, 9, 10, 12, 13, 14, 15, 16, 17, 18	4, 5, 8, 12, 13, 16, 18	
6	6	1, 2, 3, 4, 5, 6, 8, 9, 10, 12, 13, 14, 15, 16, 17, 18	6	1
7	1, 3, 4, 7, 12, 15, 16, 17	1, 2, 3, 7, 9, 10, 15	1, 3, 7, 15	
8	4, 5, 6, 8, 11, 12, 13, 16, 18	1, 2, 3, 4, 5, 8, 9, 10, 12, 13, 14, 15, 16, 17, 18	4, 5, 8, 12, 13, 16, 18	
9	1, 3, 4, 5, 6, 7, 8, 9, 10, 11, 12, 13, 14, 15, 16, 17, 18	1, 2, 3, 9, 10, 15	1, 3, 9, 10, 15	
10	1, 3, 4, 5, 6, 7, 8, 9, 10, 11, 12, 13, 14, 15, 16, 17, 18	1, 2, 3, 9, 10,15	1, 3, 9, 10, 15	
11	11	1, 2, 3, 4, 5, 8, 9, 10, 11, 12, 13, 14, 15, 16, 17, 18	11	1
12	4, 5, 6, 8, 11, 12, 13, 14, 16, 17, 18	1, 2, 3, 4, 5, 7, 8, 9, 10, 12, 13, 14, 15, 16, 17, 18	4, 5, 8, 12, 13, 14, 16, 17, 18	
13	4, 5, 6, 8, 11, 12, 13, 14, 16, 17, 18	1, 2, 3, 4, 5, 8, 9, 10, 12, 13, 14, 15, 16, 17, 18	4, 5, 8, 12, 13, 14, 16, 17, 18	
14	4, 5, 6, 8, 11, 12, 13, 14, 16, 17, 18	1, 2, 3, 9, 10, 12, 13, 14, 15, 16, 17, 18	12, 13, 14, 16, 17, 18	
15	1, 3, 4, 5, 6, 7, 8, 9, 10, 11, 12, 13, 14, 15, 16, 17, 18	1, 2, 3, 7, 9, 10, 15	1, 3, 7, 9, 10, 15	
16	4, 5, 6, 8, 11, 12, 13, 14, 16, 17, 18	1, 2, 3, 4, 5, 7, 8, 9, 10, 12, 13, 14, 15, 16, 17, 18	4, 5, 8, 12, 13, 14, 16, 17, 18	
17	4, 5, 6, 8, 11, 12, 13, 14, 16, 17, 18	1, 2, 3, 7, 9, 10, 12, 13, 14, 15, 16, 17, 18	12, 13, 14, 16, 17, 18	
18	4, 5, 6, 8, 11, 12, 13, 14, 16, 17, 18	1, 2, 3, 4, 5, 8, 9, 12, 13, 14, 15, 16, 17, 18	4, 5, 8, 12, 13, 14, 16, 17, 18	

**Table 7:** Level partitions for enablers—Iteration 2.

Enablers	Reachability Sets	Antecedent Set	Intersections	Levels
1	1, 3, 4, 5, 7, 8, 9, 10, 12, 13, 14, 15, 16, 17, 18	1, 2, 3, 7, 9, 10, 15	1, 3, 7, 9, 10, 15	
2	1, 2, 3, 4, 5, 7, 8, 9, 10, 12, 13, 14, 15, 16, 17, 18	2	2	
3	1, 3, 4, 5, 7, 8, 9, 10, 12, 13, 14, 15, 16, 17, 18	1, 2, 3, 7, 9, 15	1, 3, 7, 9, 15	
4	4, 5, 8, 12, 13, 16, 18	1, 2, 3, 4, 5, 7, 8, 9, 10, 12, 13, 14, 15, 16, 17, 18	4, 5, 8, 12, 13, 16, 18	2
5	4, 5, 8, 12, 13, 16, 18	1, 2, 3, 4, 5, 8, 9, 10, 12, 13, 14, 15, 16, 17, 18	4, 5, 8, 12, 13, 16, 18	2
7	1, 3, 4, 7, 12, 15, 16, 17	1, 2, 3, 7, 9, 10, 15	1, 3, 7, 15	
8	4, 5, 8, 12, 13, 16, 18	1, 2, 3, 4, 5, 8, 9, 10, 12, 13, 14, 15, 16, 17, 18	4, 5, 8, 12, 13, 16, 18	2
9	1, 3, 4, 5, 7, 8, 9, 10, 12, 13, 14, 15, 16, 17, 18	1, 2, 3, 9, 10, 15	1, 3, 9, 10, 15	
10	1, 3, 4, 5, 7, 8, 9, 10, 12, 13, 14, 15, 16, 17, 18	1, 2, 3, 9, 10, 15	1, 3, 9, 10, 15	
12	4, 5, 8, 12, 13, 14, 16, 17, 18	1, 2, 3, 4, 5, 7, 8, 9, 10, 12, 13, 14, 15, 16, 17, 18	4, 5, 8, 12, 13, 14, 16, 17, 18	2
13	4, 5, 8, 12, 13, 14, 16, 17, 18	1, 2, 3, 4, 5, 8, 9, 10, 12, 13, 14, 15, 16, 17, 18	4, 5, 8, 12, 13, 14, 16, 17, 18	2
14	4, 5, 8, 12, 13, 14, 16, 17, 18	1, 2, 3, 9, 10, 12, 13, 14, 15, 16, 17, 18	12, 13, 14, 16, 17, 18	
15	1, 3, 4, 5, 7, 8, 9, 10, 12, 13, 14, 15, 16, 17, 18	1, 2, 3, 7, 9, 10, 15	1, 3, 7, 9, 10, 15	
16	4, 5, 8, 12, 13, 14, 16, 17, 18	1, 2, 3, 4, 5, 7, 8, 9, 10, 12, 13, 14, 15, 16, 17, 18	4, 5, 8, 12, 13, 14, 16, 17, 18	2
17	4, 5, 8, 12, 13, 14, 16, 17, 18	1, 2, 3, 7, 9, 10, 12, 13, 14, 15, 16, 17, 18	12, 13, 14, 16, 17, 18	
18	4, 5, 8, 12, 13, 14, 16, 17, 18	1, 2, 3, 4, 5, 8, 9, 12, 13, 14, 15, 16, 17, 18	4, 5, 8, 12, 13, 14, 16, 17, 18	2

**Table 8:** Level partitions for enablers—Iteration 3.

Enablers	Reachability Sets	Antecedent Set	Intersections	Levels
1	1, 3, 7, 9, 10, 14, 15, 17	1, 2, 3, 7, 9, 10, 15	1, 3, 7, 9, 10, 15	
2	1, 2, 3, 7, 9, 10, 14, 15, 17	2	2	
3	1, 3, 7, 9, 10, 14, 15, 17	1, 2, 3, 7, 9, 15	1, 3, 7, 9, 15	
7	1, 3, 7, 15, 17	1, 2, 3, 7, 9, 10, 15	1, 3, 7, 15	
9	1, 3, 7, 9, 10, 14, 15, 17	1, 2, 3, 9, 10, 15	1, 3, 9, 10, 15	
10	1, 3, 7, 9, 10, 14, 15, 17	1, 2, 3, 9, 10, 15	1, 3, 9, 10, 15	
14	14, 17	1, 2, 3, 9, 10, 14, 15, 17	14, 17	3
15	1, 3, 7, 9, 10, 14, 15, 17	1, 2, 3, 7, 9, 10, 15	1, 3, 7, 9, 10, 15	
17	14, 17	1, 2, 3, 7, 9, 10, 14, 15, 17	14, 17	3

**Table 9:** Level partitions for enablers—Iteration 4.

Enablers	Reachability Sets	Antecedent Set	Intersections	Levels
1	1, 3, 7, 9, 10, 15	1, 2, 3, 7, 9, 10, 15	1, 3, 7, 9, 10, 15	4
2	1, 2, 3, 7, 9, 10, 15	2	2	
3	1, 3, 7, 9, 10, 15	1, 2, 3, 7, 9, 15	1, 3, 7, 9, 15	
7	1, 3, 7, 15	1, 2, 3, 7, 9, 10, 15	1, 3, 7, 15	4
9	1, 3, 7, 9, 10, 15	1, 2, 3, 9, 10, 15	1, 3, 9, 10, 15	
10	1, 3, 7, 9, 10, 15	1, 2, 3, 9, 10, 15	1, 3, 9, 10, 15	
15	1, 3, 7, 9, 10, 15	1, 2, 3, 7, 9, 10, 15	1, 3, 7, 9, 10, 15	4

**Table 10:** Level partitions for enablers—Iteration 5.

Enablers	Reachability Sets	Antecedent Set	Intersections	Levels
2	2, 3, 9, 10	2	2	
3	3, 9, 10	2, 3, 9	3, 9	
9	3, 9, 10	2, 3, 9, 10	3, 9, 10	5
10	3, 9, 10,	2, 3, 9, 10	3, 9, 10	5

**Table 11:** Level partitions for enablers—Iteration 6.

Enablers	Reachability Sets	Antecedent Set	Intersections	Levels
2	2, 3	2	2	
3	3	2, 3	3	6

**Table 12:** Level partitions for enablers—Iteration 7.

Enablers	Reachability Sets	Antecedent Set	Intersections	Levels
2	2	2	2	7

**Table 13:** Different levels of barriers to knowledge management implementation in the healthcare sector of Pakistan. Knowledge Management (KM).

Levels	Enablers
1	(6) Detection of knowledge brokers, and (11) Categorizing information to avoid overflow of information
2	(18) Knowledge filtering (16) Training and education (13) Employee empowerment (12) Employee motivation (8) Developing collaborative learning atmosphere (5) Eliminate distrust, and (4) Developing a transparent workflow
3	(14) Promotion of e-data, and (17) Hiring KM personnel’s
4	(15) Customer Feedback (7) Establishing Customer Relationship Management (CRM), and (1) Management commitment
5	(10) IT for KM, and (9) Align KM efforts with business strategy
6	(3) Long-term strategic planning
7	(2) Policy incentives
